# Unchecked immunity: a unique case of sequential immune-related adverse events with Pembrolizumab

**DOI:** 10.1186/s40425-019-0727-5

**Published:** 2019-09-12

**Authors:** N. Shah, J. Jacob, Z. Househ, E. Shiner, L. Baird, H. Soudy

**Affiliations:** 1 St George-Sutherland Basic Physician Training Network, Kogarah, New South Wales Australia; 2 Department of Anatomical Pathology, SEALS, St George Hospital, Kogarah, New South Wales Australia; 3 Department of Neurology, St George Hospital, Kogarah, New South Wales Australia; 4 Department of Aged Care, St George Hospital, Kogarah, New South Wales Australia; 5 Faculty of Medicine, University of New South Wales, Kensington, New South Wales Australia; 6 Department of Medical Oncology, St George Hospital, Kogarah, New South Wales Australia; 7 Department of Medical Oncology, The Sutherland Hospital, Caringbah, New South Wales Australia

**Keywords:** Checkpoint inhibitors, Malignant melanoma, Immune-related adverse events

## Abstract

**Background:**

Immune checkpoint inhibition has dramatically transformed the treatment of malignant melanoma. With increasing use, their unique spectrum of immune-mediated toxicity has become apparent.

**Case presentation:**

We describe a case of sequential immune-related adverse events (irAEs) in a patient with metastatic melanoma treated with single-agent anti-programmed cell death-1 (PD-1) therapy, pembrolizumab. Although numerous cases of irAEs have been reported, sequential multi-organ involvement, including progressive atopic dermatitis, vitiligo, autoimmune nephritis, autoimmune hepatitis, and autoimmune encephalitis after cessation of therapy, has not been previously documented.

**Conclusions:**

Immunosuppression resulted in clinical remission of each irAE, highlighting the importance of vigilance for autoimmune complications in patients treated with checkpoint inhibition, even after immunotherapy cessation.

## Background

Targeting of immune checkpoints is based on the natural role of specific receptors acting as negative regulators of T-cell activation. These signals play a decisive role in the maintenance of peripheral tolerance and prevention of auto-immunity [1–4]. By inhibiting these pathways, augmentation of stimulatory signals provides a means to enhance anti-tumour immune responses. The two most commonly targeted receptors include cytotoxic T-lymphocyte associated antigen 4 (CTLA-4) and programmed cell death-1 (PD-1).

Since their discovery, immune checkpoint inhibitors have transformed the treatment of numerous malignancies [5]. Consequently their list of indications has grown exponentially, as has our experience with their unique spectrum of toxicities. The non-specific immunostimulation resulting from these targeted therapies can cause a wide range of side effects in numerous organs including the skin, lungs, kidneys, gastrointestinal tract, as well as the endocrine and nervous systems [5, 6]. Many of these toxicities mimic autoimmune reactions and are commonly referred to as immune-related adverse events (irAEs). Most neurological side effects are mild (grade 1–2) and consist of non-specific symptoms such as headache, with a reported incidence of 3.8% following anti-CTLA-4 therapy, 6.1% following anti-PD-1, and 12% following combination therapy [7]. Severe neurological adverse events (grade 3–4) occur in < 1% of patients and can include a wide spectrum of syndromes including autoimmune encephalitis, aseptic meningitis, myasthenia gravis, Guillain-Barré syndrome, peripheral sensorimotor neuropathies, and posterior reversible encephalopathy syndrome [7]. One point of particular importance is that there is no direct correlation between the time of drug administration and onset of irAEs [8]. Some case reports have noted irAEs occurring weeks or even months after cessation of treatment, though the majority of complications seem to occur within the first few months of drug exposure [9].

We report a case of sequential irAEs in several distinct organ systems, including progressive atopic dermatitis, vitiligo, tubulointerstitial nephritis, autoimmune hepatitis, and a delayed-onset N-Methyl-D-Aspartate receptor antibody (NMDA-R Ig) positive encephalitis, in a man being treated for metastatic melanoma with single agent pembrolizumab.

## Presentation of CASE

**Fig. 1:**
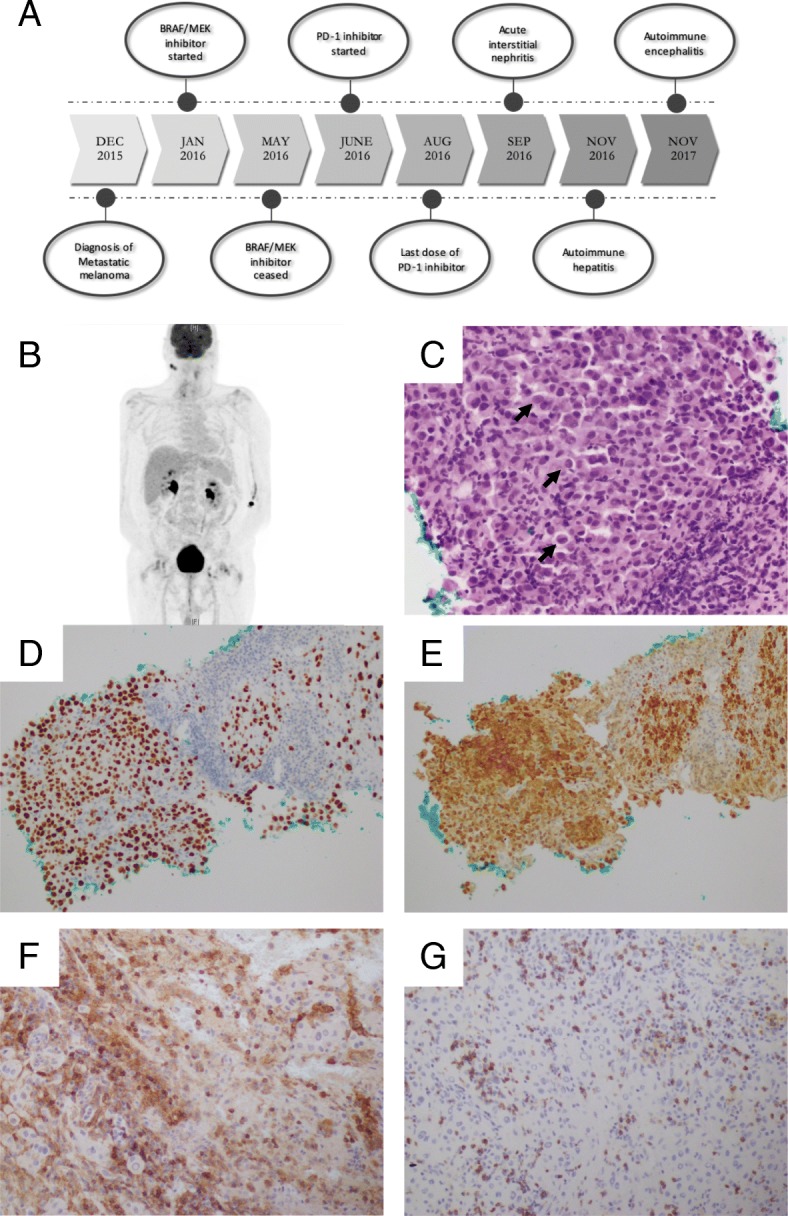
( **a** ) Timeline of diagnosis, treatment and immune-related adverse events ( **b** ) Post-treatment PET scan from April 2017: Initial L inguinal mass, as well as cerebral metastasis have resolved. Unrelated persistent bilateral parotid FDG-avidity which remained stable over serial PET scans ( **c** ) Replacement of the lymph node tissue by diffuse infiltrate of large malignant cells with occasional intranuclear inclusions (black arrows) [400x]. ( **d** ) Metastatic melanoma diagnosis confirmed by strong nuclear positivity for SoX-10 on immunohistochemical staining [200x] and ( **e** ) diffuse S-100 positivity [200x]. Immunostaining of tumour infiltrating lymphocytes showing positivity for T-cell markers ( **g** ) CD4 [200x], and ( **h** ) CD8 [200x]

A 70-year-old male, was diagnosed with metastatic melanoma in December 2015 after presenting to his general practitioner with a growing left sided inguinal mass, headaches, and constitutional symptoms, on a background of type 2 diabetes mellitus, hypertension, dyslipidaemia, a prior subsegmental left lower lobectomy for a benign mass, prior quinine-treated malaria, atopic dermatitis, and a significant smoking and drinking history. Biopsy of the inguinal mass was positive for V600E BRAF-mutant metastatic melanoma (Fig. 1). Initial Staging CT and FDG-PET scans demonstrated lesions in the left inguinal region, liver, as well as haemorrhagic lesions in his right frontal and left temporal lobes. With a normal LDH level (154 U/L), his melanoma was classified as stage 4 M1c disease. He underwent a stereotactic craniotomy and radiotherapy for the right frontal tumour, and was subsequently commenced on BRAF/MEK inhibitors (150 mg dabrafenib twice daily, and 2 mg trametinib daily). The left temporal metastases were monitored with surveillance cerebral CT scans.

Over the following four months, a significant treatment response was seen with radiological stability of the remaining two intracranial lesions, resolution of the liver lesion and metastatic iliac lymph nodes, and reducing FDG-avidity on serial PET studies. During this period, his progress was complicated by acute kidney injury, recurrent falls, delirium requiring temporary cessation of BRAF/MEK inhibitor therapy, and discharge to a low-level residential supportive care facility. Additionally, given his repeated admissions and patient preference, his treatment with dabrafenib and trametinib was ceased transitioning to a single-agent anti-PD1 therapy, with pembrolizumab (2 mg/kg every 3 weeks).

**Fig. 2:**
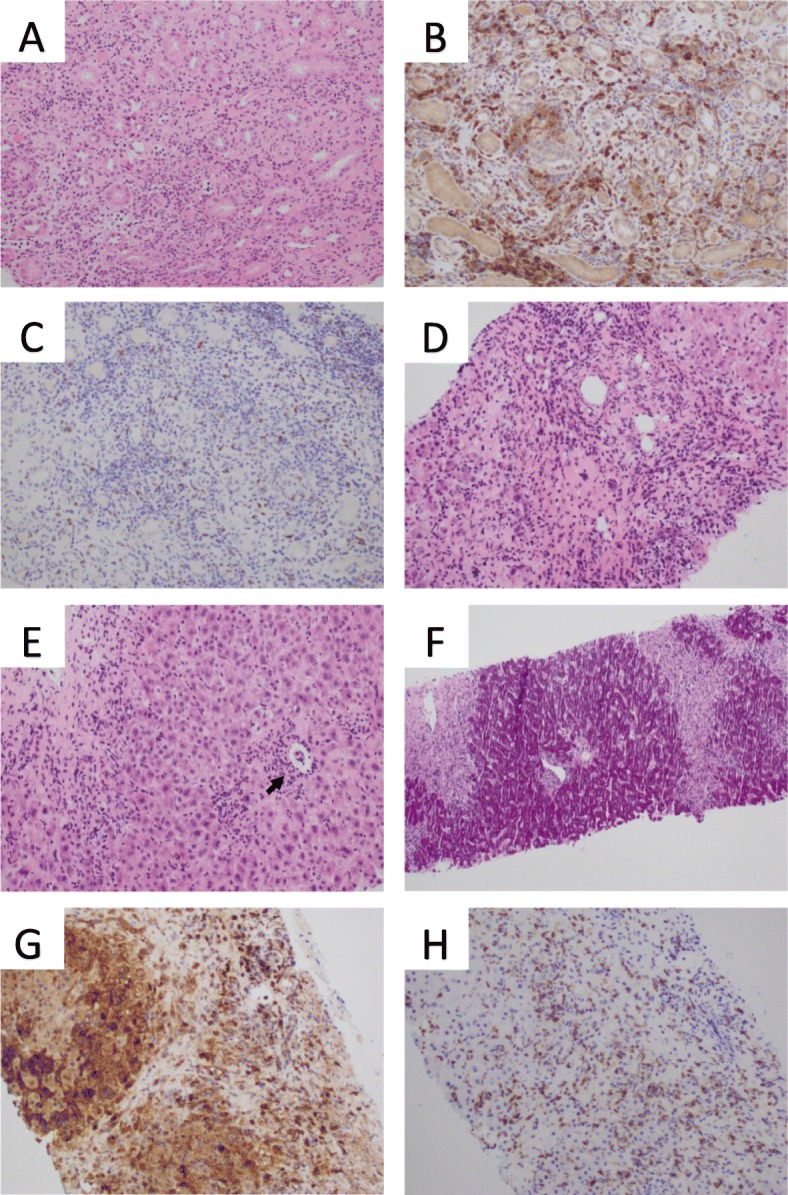
Kidney biopsy showing active tubulointerstitial nephritis ( **a** ) Interstitial inflammation with moderate numbers of eosinophils, small lymphocytes, and neutrophils [200x]. ( **b** ) Immunostaining of interstitial lymphocytes showing positivity for T-Cell markers CD4 [200x], and (c) CD8 [200x]. The liver biopsy showing features of autoimmune hepatitis process [200x] ( **d** ) Portal tract fibrosis with moderate inflammation, periportal hepatocyte ballooning, and focal necrosis. ( **e** ) Lobular activity with areas of necrosis and inflammation around central vein (black arrows) [200x] ( **f** ) Confluent necrosis highlighted by PAS stain [100X]. ( **g** ) Immunostaining of portal and periportal lymphocytes showing positivity for T-Cell markers CD4 [200x], and (H) CD8 [200x]

Initially pembrolizumab was tolerated with minimal adverse effects including transient headaches, worsening of his atopic dermatitis, and vitiligo. In the fourth cycle of treatment, he developed severe acute kidney injury (creatinine 215 mmol/L, eGFR 26 mL/min) secondary to biopsy-proven tubulointerstitial nephritis with eosinophils, consistent with a grade 3 irAE from Pembrolizumab (Fig. 2). Pembrolizumab was ceased and immunosuppression commenced with high dose oral glucocorticoids tapered over a two-month period. He had complete recovery of renal function, however, prior to reinitiating his treatment with pembrolizumab, he was re-admitted with asymptomatic abnormalities of his liver function tests (Bilirubin 80 μmol/L, ALP 534 U/L, GGT 281 U/L, ALT 1242 U/L, AST 1128 U/L). Anti-nuclear antibodies (ANA), extractable nuclear antibodies (ENA), anti-neutrophil cytoplasmic antibodies (ANCA), anti-smooth muscle antibodies, anti-mitochondrial antibodies, and liver-kidney microsomal antibodies were all negative. A liver biopsy revealed acute hepatitis with areas of centrilobular and periportal hepatocyte necrosis, consistent with autoimmune hepatitis, thought to be a delayed grade 4 irAE due to pembrolizumab (Fig. 2). Initial treatment consisted of pulsed intravenous methyl-prednisolone. Without significant improvement in hepatic function, mycophenolate was added with a transition to high-dose oral glucocorticoids, which were slowly weaned over 3 months. With biochemical resolution of the autoimmune hepatitis once off glucocorticoids, the mycophenolate was ceased after a total of 5 months of use.

In October 2017, despite ongoing remission of melanoma, a rapid deterioration occurred over 3 weeks with hypoactive delirium, recurrent falls, and brief witnessed tonic-clonic seizures, culminating in a virtually mute bed-bound state, without any focal deficits on neurological examination. A more extensive flare of atopic dermatitis was also noted with generalised erythematous, dry, and intensely pruritic skin, as well as progressive vitiligo, another notable irAE of pembrolizumab.

Magnetic resonance imaging of the brain did not show any new areas of T2 fluid attenuated inversion hyperintensity or gadolinium enhancement, and an FDG-PET scan exhibited no melanoma recurrence. Interictal electroencephalograms showed moderate generalised slowing but no epileptiform changes. Cerebrospinal fluid (CSF) showed a markedly elevated protein level (1.62 g/L) with albuminocytological dissociation, and positive NMDA receptor antibodies in both CSF and serum consistent with NMDA receptor antibody encephalitis, a fifth irAE (Grade 4), 15 months following pembrolizumab cessation. CSF cytology revealed a lymphocytosis without malignant cells, and viral PCR was negative. Serum paraneoplastic antibodies, including: anti-purkinjie cytoplasmic type 1 (Anti-Yo), anti-neuronal nuclear Type 1/2 (Anti-Hu/Anti-Ri), amiphiphysin, and PNMA2 (Ma2/Ta), were also unremarkable. Immunosuppression was again required with high-dose oral prednisone (100 mg daily) in preference to intravenous steroids due to severe mood and behavioural changes necessitating anti-psychotic therapy and uncontrolled hyperglycaemia with prior intravenous steroid therapy. With minimal cognitive improvement after 10 days, 5 days of intravenous immunoglobulin was added. His delirium slowly resolved over 4 weeks, enabling discharge to a higher care residential aged care facility with a 4 month tapering dose of prednisone. At discharge, cognitive screening demonstrated frontal and executive impairment with corresponding behavioural symptoms thought to be due to his prior frontal lobe surgery, exacerbated by high dose steroid treatment.

To date, FDG-PET and MRI brain scans continue to show complete remission of metastatic melanoma. With cessation of steroid therapy, cognitive function improved with only mild residual frontal impairments. Functional improvement in activities of daily living enabled transition back to independent living with community services.

## Methods

### Histology & immunohistochemistry

Whole formalin fixed, paraffin embedded tissue blocks underwent routine processing with hematoxylin and eosin (H&E) staining. At the time of initial reporting the lymph node core biopsy and frontal lesion were tested with immunohistochemistry for melanoma markers. Special stains were performed on kidney and liver biopsies as per department protocol. Fresh kidney biopsies were also assessed for routine direct immunofluorescence. Retrospective immunohistochemistry to further assess infiltrating inflammatory cells was performed on the frontal lesion, kidney and liver samples utilising Leica Biosystems Bond-Max autostaining (Leica Biosystems; Germany) as per manufacturer recommendation using antibodies against: CD3 (clone SP7; ThermoFisher), CD4 (clone 4B12; Novocastra); CD8 (clone C8/144B; ThermoFisher); CD68 (clone KP1; Biocare Medical) and PD-1 (clone NAT105; Biocare Medical).

## Discussion

The PD-1 receptor is expressed on B-lymphocytes and T-lymphocytes [10]. When bound by either of its two ligands, programmed death-ligand 1 or 2 (PDL-1 or PDL-2), lymphocyte proliferation, cytokine production, and survival are impaired [10]. Tumour upregulation of these ligands allows evasion from the immune system [11, 12]. By blocking this interaction, Pembrolizumab augments the immune system’s ability to recognise and destroy tumour cells, but this comes at a cost. Numerous autoimmune complications have been reported with this non-specific immune stimulation.

Dermatologic changes – including vitiligo [13], tubulointerstitial nephritis [14, 15], and autoimmune hepatitis [16] have previously been reported separately in patients treated with pembrolizumab. Additionally, a case of NMDA-R Ig encephalitis has been reported with combination PD-1 (nivolumab) and CTLA-4 (ipilimumab) therapy [17], however to our knowledge this is the first documented case of NMDA-R Ig encephalitis after single-agent pembrolizumab therapy. We postulate this patient developed multiple irAEs, including progressive atopic dermatitis, vitiligo, tubulointerstitial nephritis, autoimmune hepatitis, and an NMDA-R Ig encephalitis, triggered by immune checkpoint inhibition with pembrolizumab. The differential diagnosis in this case would be a paraneoplastic syndrome. Although a number of paraneoplastic conditions have been linked to melanoma, including hypercalcemia of malignancy [18], autoimmune cutaneous conditions [19, 20], and ocular paraneoplastic syndromes [21, 22], to our knowledge there is no association with NMDA-R Ig encephalitis.

A number of studies have suggested that autoimmunity, in the form of vitiligo, is not only common in patients receiving immunotherapy but also correlates with tumour regression. [23–26] This relatively harmless depigmentation is a result of the immune system targeting healthy melanocytes as a result of shared expression of melanocyte differentiation factors with tumour cells. One of these shared antigens is proposed to be the micropthalmia-associated transcription factor which acts as a key regulator of melanocyte survival, melanin production, and melanoma transformation. [27] Whole-exome sequencing of melanomas has also uncovered mutations in the GRIN2A gene which encodes the regulatory subunit of the NMDA receptor. [28] It is possible that NMDA-R Ig encephalitis is a consequence of molecular mimicry when the activated immune system sets off a signalling cascade creating antibodies against the NMDA-R, found on both the melanoma as well as the endogenous cells of the central nervous system. [29] The history of cerebral metastasis and prior craniotomy in this case may have created a sufficient physical disruption to the blood-brain barrier to allow peripherally-created antibodies against the NMDA-R to enter the CNS. [30]

In this case, two of the four complications occurring many months after cessation of treatment, demonstrating that checkpoint inhibition may result in long-lasting immune activation. Sequential irAEs affecting several distinct organ systems is also unusual. In this patient, it is notable that the metastatic focus in the frontal lobe had a significant CD4/CD8 lymphocytic infiltrate. Studies have suggested that tumour infiltrating lymphocytes (TILs) in melanoma are associated with better patient survival, and response to treatment [31–34]. MEK inhibitors have also been shown to increase TILs, which when combined with immunomodulatory antibodies improves the anti-tumour effects of treatment [35, 36]. This patient received BRAF/MEK inhibition prior to commencing anti-PD-1 therapy. Although the altered tumour microenvironment may have improved his response to pembrolizumab, further research is needed to determine if this contributed to the multiple sequential irAE seen in this patient and whether there is an association between TILs at the time of diagnosis and risk for irAEs. Additionally, HLA subtype testing, and further DNA genetic studies may establish if this patient has a genetic predisposition to severe irAEs.

## Conclusion

This case is unique in that it highlights the broad range of possible irAEs with checkpoint inhibition in a single patient. Although numerous cases of irAEs have been reported, to our knowledge sequential organ involvement, including progressive atopic dermatitis and a delayed NMDA-R Ig encephalitis long after cessation of anti-PD-1 therapy, has not been previously reported. Indeed this is the first case of NMDA-R Ig positive encephalitis linked to pembrolizumab. Given the temporal dichotomy between therapy cessation and symptom onset, it is important for physicians from different specialties to be aware of irAE associated with checkpoint inhibitors because treatment with immunosuppression, as demonstrated by this case study, can be highly effective in achieving autoimmune remission.

## Data Availability

Not applicable.

## Abbreviations

CSF: Cerebrospinal fluid
CTLA-4: Cytotoxic T-lymphocyte associated antigen 4
H&E: hematoxylin and eosin
irAEs: Immune-related adverse events
NMDA-R Ig: N-Methyl-D-Aspartate receptor antibody
PD-1: Programmed cell death-1
PDL-1: Programmed death-ligand 1
PDL-2: Programmed death-ligand 2
TILs: Tumour infiltrating lymphocytes
